# Lurasidone‐induced hyperosmolar hyperglycemic syndrome: A case report

**DOI:** 10.1002/npr2.12259

**Published:** 2022-05-24

**Authors:** Shota Hanyu, Yusuke Kojima, Toshiya Murai, Hirotsugu Kawashima

**Affiliations:** ^1^ Department of Psychiatry Kyoto University Hospital Kyoto Japan; ^2^ Department of Clinical Pharmacology and Therapeutics Kyoto University Hospital Kyoto Japan

**Keywords:** bipolar disorder, Drug‐related side effects and adverse reactions, Hyperglycemia, Hyperglycemic hyperosmolar nonketotic coma, Lurasidone hydrochloride.

## Abstract

**Introduction:**

Lurasidone has few metabolic adverse effects and is recommended as an alternative when other antipsychotic drugs considerably increase body weight or blood sugar concentrations.

**Case presentation:**

An 81‐year‐old man with bipolar disorder developed hyperosmolar hyperglycemic syndrome as a side effect of lurasidone. Routine monitoring of blood glucose concentrations led to the early detection and treatment of this disease, preventing life‐threatening complications.

**Discussion and conclusion:**

We describe a rare case of lurasidone‐induced hyperosmolar hyperglycemic syndrome. The mortality rate of this syndrome is estimated to be up to 20%. This rate is significantly higher than that of diabetic ketoacidosis (currently <2%). Although lurasidone is considered to have a low risk of raising blood glucose concentrations, symptoms of hyperglycemia must be evaluated and blood glucose concentrations should be monitored regularly.

## 1 INTRODUCTION

Typical antipsychotics were first developed in the 1950s and have been used to treat psychosis. However, their side effects, such as extrapyramidal symptoms, hyperprolactinemia, negative symptoms, and cognitive decline, have been considered to be problematic. Atypical antipsychotics are commonly used because they have fewer extrapyramidal symptoms than typical antipsychotics, and are effective in improving symptoms of schizophrenia and bipolar disorder. Side effects of these atypical antipsychotics include impaired glucose tolerance and weight gain. In some cases, the onset of diabetes and ketoacidosis may force patients to stop taking these drugs.

Lurasidone is categorized as an atypical antipsychotic and is effective for patients with acute bipolar I depression and schizophrenia. Common side effects of lurasidone include nausea, akathisia, drowsiness, and vomiting. Because lurasidone is considered to have fewer metabolic adverse effects than other antipsychotics, it is recommended as an alternative when other antipsychotics considerably increase body weight or blood sugar concentrations.^1^

We report here a rare case of hyperosmolar hyperglycemic syndrome as a side effect of lurasidone. Because hyperosmolar hyperglycemic syndrome has a high mortality rate, its early detection and treatment are critical.

## 2 CASE PRESENTATION

The patient was an 81‐year‐old man. He was diagnosed with bipolar disorder at the age of 67 years. He had been hospitalized on numerous occasions for the treatment of mania and depression. Because his depression had deteriorated and he had suicidal tendencies, he was hospitalized again (day 1). Asenapine maleate had been used as maintenance therapy before admission. No obvious side effects were observed, including elevated blood glucose levels.

At the age of 70 years, he suffered from volvulus of the stomach, which caused widespread ischemic gastritis. As a result, his pylorus narrowed and he was unable to orally ingest food. Consequently, he had been fed enterally through an intestinal fistula. He was taking insulin and oral hypoglycemic medication for the treatment of diabetes. Because lamotrigine (200 mg/day) was not fully effective for the depressive symptoms, he additionally started to take lurasidone (10 mg/day) on day 30. On day 34, the dosage of lurasidone was increased to 20 mg/day. At that time, he received 1240 ml/day of tube feeding (including 1016 ml of water and 1500 kcal). On day 37, he vomited and his level of consciousness declined. The patient’s vital signs were a body temperature of 36.7°C, blood pressure of 123/65 mmHg, and pulse rate of 158 beats/min. The results of blood tests and urinalysis on admission (day 1) and day 37 are shown in Table 1. Head and abdominal computed tomographic scans showed no intracranial or trunk lesions that could cause these symptoms.

**TABLE 1:** Tests on days 1 and 37

Blood test	Day 1	Day 37	
Osmolarity		343	mOsm/kg
pH		7.46	
HCO_3_		29.8	mmol/L
Glucose	104	698	mg/dL
WBC	7310	9580	/μL
Hemoglobin	14.1	12.1	g/dL
Platelet	19.3	21	×10^4^/μL
LDH	199	156	U/L
BUN	19	44	mg/dL
AST	30	19	IU/L
ALT	19	16	IU/L
ALP	148	164	IU/L
γ‐GTP	14	19	IU/L
CRP	0.3	0.1>	mg/dL
Total protein	7.4	6.2	g/dL
Albumin	4.2	3.2	g/dL
Total bilirubin	0.4	0.3	mg/dL
Creatinine	0.66	0.68	mg/dL
eGFR	86.6	83.8	ml/min/1.73m^2^
Na	139	139	mEq/L
K	3.8	5	mEq/L
Cl	100	102	mmol/L
Ca	9.1	8.7	mg/dL
Urinalysis	day1	day37	
Ketone bodies		(−)	
Pyuria		(−)	

Abbreviations: ALP, alkaline phosphatase; ALT, alanine aminotransferase; AST, aspartate aminotransferase; BUN, blood urea nitrogen; Ca, calcium; Cl, chloride; CRP, C‐reactive protein; eGFR, estimated glomerular filtration rate; HCO_3_, bicarbonate ion; K, potassium; LDH, lactate dehydrogenase; Na, sodium; pH, potential hydrogen; WBC, white blood cell; γ‐GTP, γ‐glutamyl transpeptidase.

On the basis of these findings, he was diagnosed with hyperosmolar hyperglycemic syndrome. The administration of lurasidone was stopped. Intravenous fluid supplementation was started to treat dehydration, and the insulin dosage was adjusted to lower blood glucose concentrations. After his blood glucose concentrations and osmotic pressure normalized, he recovered from hyperosmolar hyperglycemic syndrome the next day. Adjusting the dosage of the enteral nutrition agent led to a further improvement in blood sugar control.

## 3 DISCUSSION AND CONCLUSION

Lurasidone is considered unlikely to cause hyperglycemia or diabetes. In addition, studies in adults have shown that switching from an antipsychotic drug with a high risk of metabolic syndrome to lurasidone can improve blood glucose concentrations and other metabolism‐related parameters.^1^ In this case, there was no change in the enteral nutrition dose before and after the start of lurasidone administration. However, blood glucose concentrations increased after the patient started taking lurasidone. Therefore, the onset of this syndrome may have been triggered by the administration of lurasidone. There are few, if any, cases of hyperosmolar hyperglycemic syndrome with the simultaneous use of lurasidone as in our case.

Generally, antipsychotic‐induced hyperglycemia is considered to result from insulin resistance and β‐cell damage. Insulin resistance is mainly triggered in two ways. First, antipsychotics can block the insulin signaling pathways of target cells, such as muscle cells, hepatocytes, and fat cells, causing insulin resistance. Second, antipsychotic‐induced obesity causes inflammation and high concentrations of free fatty acids, which can lead to insulin resistance.^2^ Obesity is caused by an increased appetite due to effects on the feeding center through the blockade of serotonin 5‐HT2C and histamine H1 receptors.^3^ However, lurasidone has low affinities for these receptors. β‐cell damage can be caused by antipsychotics directly, leading to dysfunction and apoptosis of β‐cells.^2^ This syndrome can be triggered by the pathway above, although the pathophysiological mechanism of lurasidone‐induced hyperosmolar hyperglycemic syndrome has not been determined and further research is required.

Hyperosmolar hyperglycemic syndrome is a metabolic complication of diabetes mellitus, and is characterized by hyperglycemia, severe dehydration, hyperosmolarity, and a disturbance in consciousness. This syndrome commonly occurs in type 2 diabetes. The diagnosis of hyperosmolar hyperglycemic syndrome is based on hyperosmolarity, hyperglycemia, and a lack of ketosis.^4^ This syndrome is treated with intravenous saline and insulin therapy.^5^ The estimated mortality rate of hyperosmolar hyperglycemic syndrome is up to 20%, which is significantly higher than the mortality rate of diabetic ketoacidosis (currently <2%).^4^, ^6^ Hyperosmolar hyperglycemic syndrome is caused by relative insulin deficiency accompanied by an increase in blood concentrations of counterregulatory hormones, which leads to gluconeogenesis and glycogenolysis. As a result, hyperglycemia occurs, and this triggers polyuria resulting from glucose‐induced osmotic diuresis. Polyuria causes electrolyte abnormalities and dehydration, which finally leads to hyperosmolar hyperglycemic syndrome.^7^

In this case, the patient regularly had blood glucose measurements since admission. Fortunately, routine monitoring of blood glucose concentrations led to the early detection of hyperosmolar hyperglycemic syndrome, which resulted in saving his life. Because hyperosmolar hyperglycemic syndrome has a high mortality rate, blood tests need to be performed in patients taking antipsychotic drugs on a regular basis. Blood glucose concentrations should be measured each time a hyperglycemic symptom, such as a dry mouth, polyuria, or impaired consciousness, is observed. Although lurasidone is considered to have a low risk of raising blood glucose concentrations, its use should not preclude regular blood glucose monitoring.

## AUTHOR CONTRIBUTIONS

SH, YK, and HK treated the patient. SH drafted the manuscript. YK, HK, and TM critically reviewed the draft and revised it. All authors read and approved the final manuscript.

## CONFLICT OF INTEREST

The authors declare no conflict of interest.

### APPROVAL OF THE RESEARCH PROTOCOL BY AN INSTITUTIONAL REVIEWER BOARD

Not applicable.

### INFORMED CONSENT

Written informed consent was obtained from the patient for the publication of this case report.

### REGISTRY AND THE REGISTRATION NO. OF THE STUDY/TRIAL

Not applicable.

### ANIMAL STUDIES

Not applicable.

## Data Availability

Data sharing is not applicable for this article as no datasets were generated or analyzed during the current study.
